# Reinstatement of the corticioid genus *Leifia* (Hymenochaetales, Basidiomycota) with a new species *L.brevispora* from Hubei, Central China

**DOI:** 10.3897/mycokeys.51.33262

**Published:** 2019-05-08

**Authors:** Shi-Liang Liu, Yusufjon Gafforov, Xian-Ying Zhang, Hong-Ling Wang, Xue-Wei Wang, Li-Wei Zhou

**Affiliations:** 1 State Key Laboratory of Mycology, Institute of Microbiology, Chinese Academy of Sciences, Beijing 100101, China; 2 Institute of Applied Ecology, Chinese Academy of Sciences, Shenyang 110016, China; 3 Laboratory of Mycology, Institute of Botany, Academy of Sciences of the Republic of Uzbekistan, 32 Durmon yuli Street, Tashkent, 100125, Uzbekistan; 4 College of Life Engineering, Shenyang Institute of Technology, Fushun 113122, China; 5 University of Chinese Academy of Sciences, Beijing 100049, China

**Keywords:** Morphology, *
Odonticium
*, phylogeny, taxonomy, wood-inhabiting fungi, 1 new taxon

## Abstract

The monotypic genus *Leifia* was previously considered to be a later synonym of *Odonticium*. With the morphological and phylogenetic evidence provided by an additional four East Asian specimens, we propose to reinstate *Leifia* as an independent genus in Hymenochaetales. *Leifia* morphologically differs from *Odonticium* by its grandinioid hymenophore with hyphal strands, numerous thick-walled cystidia with an invaginated apical end and narrowly and thick-walled basidia. The phylogeny generated from the current data set of ITS and 28S regions indicates that *Leifia* forms a sister clade to *Odonticium*. Besides the generic type *Leifiaflabelliradiata* in the *Leifia* clade, two specimens, collected from Hubei, Central China, are newly introduced as *Leifiabrevispora*. This new species is the second species of *Leifia* and differs from the generic type by its shorter basidiospores and distribution in warm-temperate to subtropical areas in East Asia. The additional two specimens, collected from Da Lat, Viet Nam, differ morphologically, both from each other and from known species of *Leifia*, but more samples need to be examined before further taxonomic decisions can be made.

## Introduction

*Leifia* Ginns is a monotypic genus of wood-inhabiting basidiomycetes introduced by Ginns (1998). The basionym of its type is *Phanerochaeteflabelliradiata* J. Erikss. & Hjortstam that was described from Norway (Eriksson et al. 1981). Burdsall (1985) regarded *P.flabelliradiata* as a deviating element in *Phanerochaete* P. Karst. and transferred it to *Tubulicrinis* Donk. Hjortstam (1986) accepted the concept of *Phanerochaete* sensu Burdsall (1985), but he considered that *Tubulicrinisflabelliradiatus* (J. Erikss. & Hjortstam) Burds. did not fit the concept of *Tubulicrinis* or any other known genus and thus erected a new genus *Granulocystis* Hjortstam to accommodate this species. Unfortunately, *Granulocystis* is an illegitimate later homonym for *Granulocystis* Hindák, a genus of green algae (Code of Nomenclature Art. 53.1, Turland 2018). Therefore, Ginns (1998) introduced *Leifia* replacing *Granulocystis*. By examining Russian specimens of *Leifiaflabelliradiata* (J. Erikss. & Hjortstam) Ginns, Zmitrovich (2001) combined this species to *Odonticium* Parmasto as *O.flabelliradiatum* (J. Erikss. & Hjortstam) Zmitr. that is the currently accepted name of this species in MycoBank and Index Fungorum. Correspondingly, *Leifia* is treated as a synonym of *Odonticium*.

Till now, Larsson et al. (2006) is the single paper which includes the species *Odonticiumflabelliradiatum* in a phylogenetic analysis. Although *Odonticiumflabelliradiatum* grouped with *O.romellii* (S. Lundell) Parmasto, the generic type of *Odonticium* and two species of *Repetobasidium* J. Erikss. with a full Bayesian posterior probability (BPP) support in the *Rickenella* Raithelh. clade of Hymenochaetales, Larsson et al. (2006) considered that this clade might not be reliable due to the lack of morphological similarities and still used the name *Leifiaflabelliradiata* rather than *O.flabelliradiatum*. However, no further taxonomic opinion relating to *Leifia* was provided in Larsson et al. (2006).

In 2017, four specimens close to *Odonticiumflabelliradiatum* were collected from Central China and Vietnam, which draw our attention to the taxonomic status and diversity of *Leifia*. Based on morphological and molecular evidence, we propose the reinstatement of *Leifia* and reveal a higher diversity of this genus.

## Materials and methods

Specimens studied are deposited in the herbarium of Institute of Applied Ecology, Chinese Academy of Sciences (IFP). Morphological photos were taken with a digital camera Canon E12 (Tokyo, Japan) in the field. Morphological observations were made with Nikon SMZ 645 and SMZ 1000 stereomicroscopes and a Nikon Eclipse 80i light microscope (Tokyo, Japan) at magnifications up to 1000×. Microscopic procedures followed Hjortstam et al. (1987). Basidiocarp sections were prepared in Melzer’s reagent, lactic acid Cotton Blue (CB) and 3% potassium hydroxide (KOH). All microscopic measurements were made in CB. When presenting the variation of basidiospore sizes, 5% of the measurements were excluded from each end of the range and are given in parentheses. The following abbreviations are used in the text: L = mean basidiospore length (arithmetic average of all measured basidiospores), W = mean basidiospore width (arithmetic average of all measured basidiospores), Q = variation in the L/W ratios between the specimens studied, n (a/b) = number of spores (a) measured from given number (b) of specimens.

The four specimens newly collected were subjected to polymerase chain reaction (PCR) directly with the Phire Plant Direct PCR kit (Finnzymes Oy, Espoo, Finland), following the manufacturer’s instructions. The nuc rDNA ITS1-5.8S-ITS2 (ITS barcode) and 28S regions were amplified using the primer pairs ITS1-F (Gardes and Bruns 1993) or ITS5 and ITS4 (White et al. 1990) and LR0R and LR7 (Vilgalys and Hester 1990), respectively. The PCR procedure was as follows: initial denaturation at 98°C for 5 min, followed by 39 cycles at 98 °C for 5 s, 59 °C for 5 s (ITS region)/48 °C for 5 s (28S region) and 72 °C for 5 s, with a final extension at 72 °C for 10 min. The PCR products were sequenced at the Beijing Genomics Institute, China, with the same primers used for PCR. All newly generated sequences were deposited in GenBank (Table 1).

The current dataset for phylogenetic analysis was mainly adopted from Larsson et al. (2006), where, to avoid redundance, taxa in the *Rickenella* clade including *Leifiaflabelliradiata* were mostly referred to, while taxa in other clades were representatively selected (Table 1). *Sistotremabrinkmannii* (Bres.) J. Erikss. was selected as an outgroup taxon. Besides taxa in Hymenochaetales, *Protodontiapiceicola* (Kühner ex Bourdot) G.W. Martin and *Exidiopsiscalcea* (Pers.) K. Wells from Auriculariales were also included as additional ingroup taxa. The ITS and 28S datasets were separately aligned with MAFFT 7.110 (Katoh and Standley 2013) with the G-INI-I option (Katoh et al. 2005) and then the two resulting alignments were concatenated as a single alignment deposited in TreeBASE (study no. 23768). The best-fit evolutionary model for this concatenated alignment was estimated as GTR+I+G with jModel Test (Guindon and Gascuel 2003; Posada 2008). Maximum likelihood (ML) and Bayesian Inference (BI) methods were conducted to perform phylogenetic analysis, respectively, using raxmlGUI 1.2 (Silvestro and Michalak 2012; Stamatakis 2006) and MrBayes 3.2 (Ronquist et al. 2012). In the ML analysis, bootstrap (BS) values were tested under the auto FC option (Pattengale et al. 2010). In the BI analysis, two independent runs were employed. Each run had four chains of 10 000 000 generations and started from random trees. Chain convergence was determined with Tracer 1.5 (http://tree.bio.ed.ac.uk/software/tracer/). After sampling every 1000th generation, the first 25% of sampled trees was removed, whereas the other 75% was subjected to construction of a 50% majority consensus tree and calculation of BPPs. The ML and BI methods generated congruent topologies in main lineages. Therefore, the topology generated in the ML analysis is presented and the BS values and BPPs, simultaneously above 50% and 0.7, respectively, are shown at the nodes.

To further differentiate the taxa of *Leifia*, the distance matrix of the alignment of their ITS sequences (5.8S and ITS2 region) were estimated using MEGA5 (Tamura et al. 2011) under the parameters of maximum composite likelihood model, uniform rates amongst sites and pairwise deletion of gaps/missing data treatment.

**Table 1.** Specimens used for the phylogenetic analyses.

**Species ** ^a^	**Voucher/strain number**	**GenBank accession number**		**Sequence reference**	**Origin**
		**ITS**	**LSU**		
* Atheloderma mirabile *	TAA 169235	DQ873592	DQ873592	Larsson et al. (2006)	Estonia
* Basidioradulum radula *	AFTOL-ID 451	DQ234537	AY700184	Unpublished	unknown
* Blasiphalia pseudogrisella *	Lutzoni 930728-3	U66437	U66437	Lutzoni (1997)	unknown
* Coltricia perennis *	DSH 93-198	DQ234559	AF287854	Hibbett et al. (2000)	unknown
* Coniferiporia weirii *	JV 0407/8J	KR350569	KR350557	Zhou et al. (2016)	USA
* Cylindrosporus flavidus *	Dai 13213	KP875564	KP875561	Zhou (2015)	China
* Cyphellostereum laeve *	JJ 020909	EU118621	EU118621	Larsson (2007a)	Sweden
* Exidiopsis calcea *	KHL 11075	AY463406	AY586654	Larsson et al. (2004)	Sweden
* Fomitiporella caryophylli *	CBS 448.76	AY558611	AY059021	Wagner and Fischer (2002); Jeong et al. (2005)	India
* Fomitiporia hartigii *	CBS 162.30	AY558621	AF311005	Jeong et al. (2005)	Russia
* Fulvifomes fastuosus *	CBS 213.36	AY558615	AY059057	Jeong et al. (2005)	Philippines
* Fulvoderma scaurum *	LWZ 20130909-2	MF860780	MF860731	Zhou et al. (2018a)	China
* Globulicium hiemale *	Hjm 19007	DQ873595	DQ873595	Larsson et al. (2006)	Sweden
* Hymenochaete adusta *	CBS 759.91	AY558594	AF385161	Jeong et al. (2005)	Unknown
* Hyphoderma capitatum *	KHL 8464 (GB)	DQ677491	DQ677491	Larsson (2007b)	Sweden
* Hyphoderma orphanellum *	NH 12208 (GB)	DQ677500	DQ677500	Larsson (2007b)	Russia
* Hyphoderma sibiricum *	KHL 4141 (GB)	DQ677503	DQ677503	Larsson (2007b)	Sweden
* Hyphodontia alutaria *	KHL 11889	DQ873603	DQ873603	Larsson et al. (2006)	Sweden
* Hyphodontia arguta *	Hjm 18726	DQ873605	DQ873605	Larsson et al. (2006)	Sweden
*Hyphodontia* sp.	H Berglund 1117	DQ873633	DQ873634	Larsson et al. (2006)	Sweden
* Kneiffiella abieticola *	KHL 12498	DQ873601	DQ873601	Larsson et al. (2006)	Sweden
* Kneiffiella barba-jovis *	KHL 11730	DQ873609	DQ873610	Larsson et al. (2006)	Sweden
* Kneiffiella curvispora *	KHL	DQ873615	DQ873616	Larsson et al. (2006)	Finland
* Kneiffiella floccosa *	Berglund 150-02	DQ873618	DQ873618	Larsson et al. (2006)	Sweden
* Leifia brevispora *	LWZ 20170820-46	MK343469	MK343473	This study	China
* Leifia brevispora *	LWZ 20170820-48	MK343470	MK343474	This study	China
* Leifia flabelliradiata *	KG Nilsson 36270	DQ873635	DQ873635	Larsson et al. (2006)	Sweden
*Leifia*sp. 1	LWZ 20171015-36	MK343471	MK343475	This study	Vietnam
*Leifia* sp. 2	LWZ 20171015-38	MK343472	MK343476	This study	Vietnam
* Loreleia marchantiae *	Lutzoni 930826-1	U66432	U66432	Lutzoni (1997)	unknown
* Lyomyces crustosus *	KHL 11731	DQ873614	DQ873614	Larsson et al. (2006)	Finland
* Lyomyces griseliniae *	KHL 12971 (GB)	DQ873651	DQ873651	Larsson et al. (2006)	Costa Rica
* Lyomyces pruni *	Ryberg 021018	DQ873624	DQ873625	Larsson et al. (2006)	Sweden
*Odonticiumromellii*1	H 6059319	MF319073	MF318929	Korotkin (2017)	Finland
*Odonticiumromellii* 2	KHL s. n.	DQ873639	DQ873639	Larsson et al. (2006)	Norway
* Palifer verecundus *	KHL 12261 (GB)	DQ873642	DQ873643	Larsson et al. (2006)	USA
* Peniophorella praetermissum *	KHL 13164 (GB)	DQ873597	DQ873597	Larsson et al. (2006)	Estonia
* Peniophorella puberum *	KHL 13154 (GB)	DQ873599	DQ873599	Larsson et al. (2006)	Estonia
* Protodontia piceicola *	KHL 11763 (GB)	DQ873660	DQ873660	Larsson et al. (2006)	Sweden
* Repetobasidium conicum *	KHL 12338	DQ873647	DQ873647	Larsson et al. (2006)	USA
*Rickenellafibula* 1	AD86033	AY463464	AY586710	Larsson et al. (2004)	Sweden
*Rickenellafibula* 2	TENN 071482	MF319083	MF318943	Korotkin (2017)	USA
* Rickenella mellea *	Lamoure 74-20h 1/9.91	U66438	U66438	Lutzoni (1997)	unknown
* Rigidoporus corticola *	KHL 13217 (GB)	DQ873641	DQ873641	Larsson et al. (2006)	Estonia
* Sidera lunata *	JS 15063	DQ873593	DQ873593	Larsson et al. (2006)	Norway
* Sistotrema brinkmannii *	KHL 14078 (GB)	KF218967	KF218967	Larsson and Kotiranta (2013)	Sweden
* Skvortzovia furfuraceum *	KHL 11738 (GB)	DQ873648	DQ873648	Larsson et al. (2006)	Finland
* Skvortzovia furfurella *	KHL 10180 (GB)	DQ873649	DQ873649	Larsson et al. (2006)	Puerto Rico
* Skvortzovia georgica *	KHL 12019 (GB)	DQ873645	DQ873645	Larsson et al. (2006)	Norway
* Skvortzovia pinicola *	KHL 12224 (GB)	DQ873637	DQ873637	Larsson et al. (2006)	USA
* Sphaerobasidium minutum *	KHL 11714	DQ873652	DQ873653	Larsson et al. (2006)	Finland
* Sphagnomphalia revibasidiata *	Lutzoni 930826-1	U66441	U66441	Lutzoni (1997)	unknown
* Trichaptum abietinum *	NH 12842 (GB)	AF347104	AF347104	Larsson et al. (2004)	Finland
* Tubulicrinis globisporus *	KHL 12133	DQ873655	DQ873655	Larsson et al. (2006)	Sweden
* Tubulicrinis hirtellus *	KHL 11717 (GB)	DQ873657	DQ873657	Larsson et al. (2004)	Finland
* Tubulicrinis inornatus *	KHL 11763 (GB)	DQ873659	DQ873659	Larsson et al. (2004)	Finland
* Tubulicrinis subulatus *	KHL11079	AY463478	AY586722	Larsson et al. (2004)	Sweden
* Xylodon asperus *	KG Nilsson s. n.	DQ873606	DQ873607	Larsson et al. (2006)	Sweden
* Xylodon brevisetus *	KHL 12386	DQ873612	DQ873612	Larsson et al. (2006)	Sweden
* Xylodon detriticus *	K.G. Nilsson 990902	DQ677507	DQ677507	Larsson (2007b)	Sweden
* Xylodon nespori *	B Nordon 030915	DQ873622	DQ873622	Larsson et al. (2006)	Sweden
* Xylodon rimosissimus *	Ryberg 021031 (GB)	DQ873627	DQ873628	Larsson et al. (2006)	Sweden

^a^Species names are adopted from recent taxonomic proposals.

## Results

From four studied specimens, four ITS and four 28S sequences were newly generated (Table 1). These sequences were incorporated in the dataset of Larsson et al. (2006) with an emphasis of taxa in the *Rickenella* clade. The current dataset included 62 taxa, each with an ITS and a 28S sequence. The concatenated alignment had 2426 characters. The BS search in the ML analysis stopped after 350 replicates. In the BI analysis, all chains were converged as suggested by the effective sample sizes of all parameters above 3300 and by the potential scale reduction factors close to 1000.

**Figure 1.**
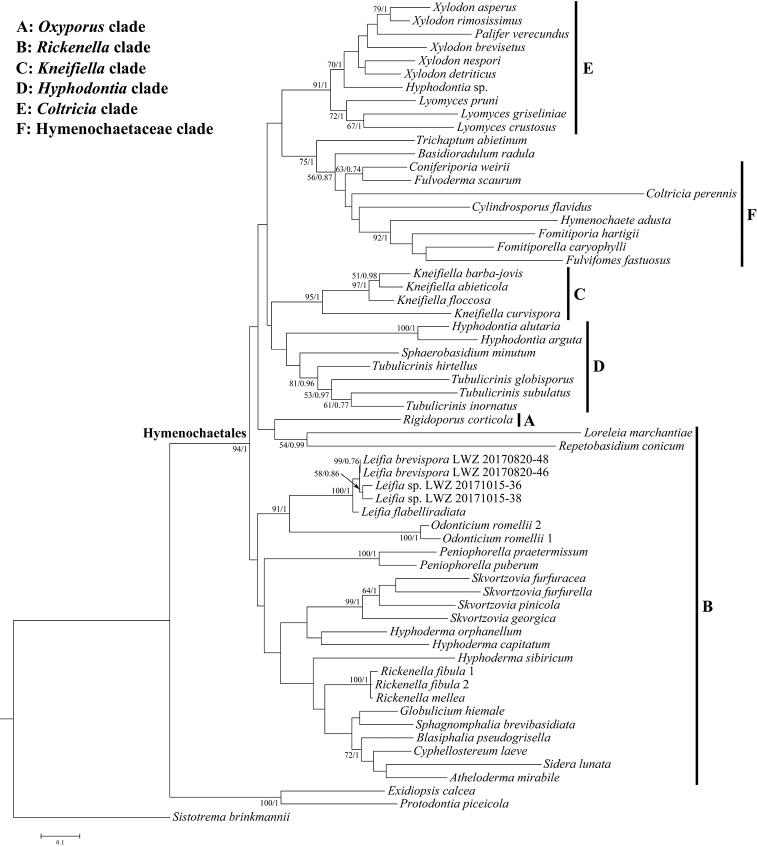
Phylogenetic relationship between *Odonticiumromellii* and *Leifia*, based on the concatenated dataset of ITS and 28S regions. The topology was generated from the maximum likelihood analysis and the bootstrap values and Bayesian posterior probability, simultaneously above 50% and 0.7, respectively, are shown at the nodes. The clade names are adapted from Larsson et al. (2006) and the species names from recent taxonomic proposals.

The current phylogeny (Figure 1) recovered Hymenochaetales as a strongly supported clade (94%, 1.00). Amongst Hymenochaetales, the *Oxyporus* (Bourdot & Galzin) Donk clade, the *Kneiffiella* P. Karst. clade, the *Hyphodontia* J. Erikss. clade and the Hymenochaetaceae clade were recovered like those in Larsson et al. (2006), although the latter two clades received no statistical support (Figure 1). The so-called *Coltricia* Gray clade in Larsson et al. (2006) here consisted entirely of corticioid species currently referred to *Lyomyces* P. Karst., *Palifer* Stalpers & P.K. Buchanan and *Xylodon* (Pers.) Gray, while *Coltriciaperennis* (L.) Murrill nested within the Hymenochaetaceae clade (Figure 1). The *Rickenella* clade of Larsson et al. (2006), the focus group for this study, did not group together well, but *Odonticiumromellii* and *Leifiaflabelliradiata* formed a strongly supported clade (91%, 1.00; Figure 1) like that in Larsson et al. (2006). The four newly sequenced specimens, also in this clade, had a closer relationship with *L.flabelliradiata* (100%, 1.00; Figure 1) than with *Odonticium*. Besides the lack of morphological similarities between *Odonticium* and *Leifia*, the branch length separating *Odonticium* from *Leifia* and related taxa also indicated that the two genera should be treated as independent.

In the *Leifia* clade, four newly sequenced specimens formed two subclades: *LWZ 20170820-46* and *LWZ 20170820-48* (99%, 0.76) and *LWZ 20171015-36* and *LWZ 20171015-38* (58%, 0.86), which were both separated from *L.flabelliradiata*. The distance matrix of ITS sequences (Table 2) indicated that *LWZ 20171015-36* and *LWZ 20171015-38* represented two distinct lineages (4.4%), while *LWZ 20170820-46* and *LWZ 20170820-48* represented one lineage distinctly different from *LWZ 20171015-36* (3.5%) and *LWZ 20171015-38* (2.9%) and moderately from *L.flabelliradiata* (1.3%).

**Table 2.** Distance matrix of the alignment of ITS sequences (5.8S and ITS2 region) from *Leifia* specimens.

	**Species**	**1**	**2**	**3**	**4**	**5**
1	* L. flabelliradiata *					
2	*L.brevispora* (LWZ 20170820-46)	0.013				
3	*L.brevispora* (LWZ 20170820-48)	0.013	0.000			
4	*L.* sp. (LWZ 20171015-36)	0.043	0.035	0.035		
5	*L.* sp. (LWZ 20171015-38)	0.036	0.029	0.029	0.044	

## Taxonomy

### Leifia brevispora

Taxon classificationFungiCorticialesCorticiaceae

Gafforov, S.L. Liu & L.W. Zhou sp. nov.

829252

Figures 2 and 3

#### Diagnosis.

The species is distinct from *Leifiaflabelliradiata* by shorter basidiospores and by being distributed in warm-temperate to subtropical areas in East Asia.

#### Typification.

CHINA. Hubei Province, Wudangshan Town, Wudangshan National Forest Park, on fallen angiosperm branch, 20 Aug 2017, *LWZ 20170820-46* (**holotype** in IFP 019239). GenBank: ITS = MK343469; 28S = MK343473.

#### Etymology.

*brevispora* (Latin), referring to short basidiospores.

#### Basidiomata.

Annual, resupinate, inseparable from substrate, effused, up to 0.6 mm thick. Hymenophore grandinioid to subodontioid. Margin white, smooth or minutely fibrous, sometimes bearing hyphal strands, thinning out, up to 2 mm wide. Aculei cream to buff in colour, rounded to ellipsoid, 2–3 per mm, up to 0.5 mm long, several being clustered together when dry. Subiculum white, up to 100 μm thick.

#### Microscopic structures.

Hyphal system monomitic; generative hyphae without clamp connections. Subicular hyphae hyaline, thin- to thick-walled, occasionally branched, frequently septate, more or less parallel to substrate, 2–4 μm wide. Aculeus (subhymenial) hyphae hyaline, distinctly thick-walled, mainly vertically intertwined, 2–4 μm wide. Cystidia hyaline, thick-walled, tubular with an invaginated apical end, 60–100 × 5–7 μm, swelling in KOH. Basidia hyaline, thick-walled, clavate to cylindrical, with four sterigmata each 2–3 μm long and a simple septum at the base, 14–18 × 4.5–5.5 μm. Basidioles similar in shape to basidia, but smaller. Basidiospores ellipsoid, hyaline, thin-walled, smooth, inamyloid and indextrinoid, acyanophilous, 3.8–4.5(–5) × (1.8–)2–2.5 μm, L = 4.13 μm, W = 2.14 μm, Q = 1.92–1.96 (60/2).

#### Other specimen examined.

CHINA. Hubei Province, Wudangshan Town, Wudangshan National Forest Park, on fallen angiosperm branch, 20 Aug 2017, *LWZ 20170820-48* (IFP 019240).

#### Notes.

The grandinioid hymenophore, simple-septate hyphae, distinctly thick-walled cystidia with an invaginated apical end and ellipsoid to subovate basidiospores with a straight or concave side, indicate that the new species is the second member of *Leifia*. Moreover, the phylogeny inferred from the ITS and 28S dataset also confirm the taxonomic position of *L.brevispora*. The generic type of *Leifia*, *L.flabelliradiata*, differs from *L.brevispora* by having longer basidiospores (4.5–5.5 × 2–2.5 µm) and a distribution in Europe (Eriksson et al. 1981).

**Figure 2.**
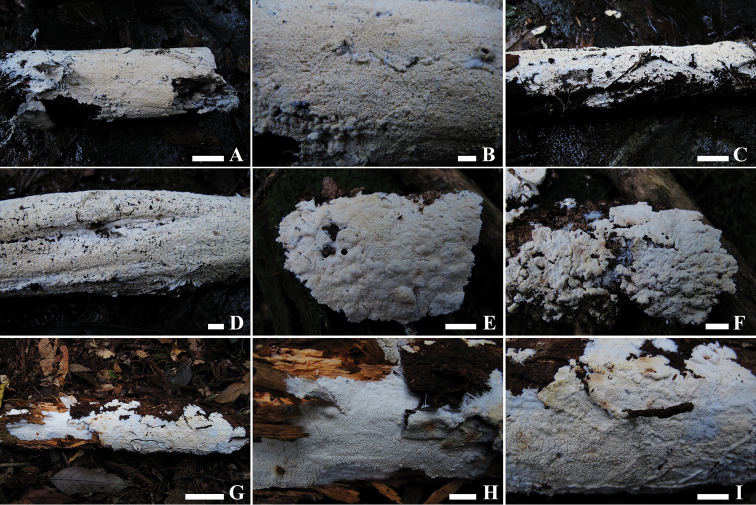
Basidiocarps of *Leifia* in situ. A-B. *L.brevispora* (LWZ 20170820-46, holotype). C-D. *L.brevispora* (LWZ 20170820-48, paratype). E-F. *Leifia* sp. (LWZ 20171015-36). G-I. *Leifia* sp. (LWZ 20171015-38). Scale bars: A, C, G: 5 cm; B, D−F, H−I: 1 cm.

**Figure 3.**
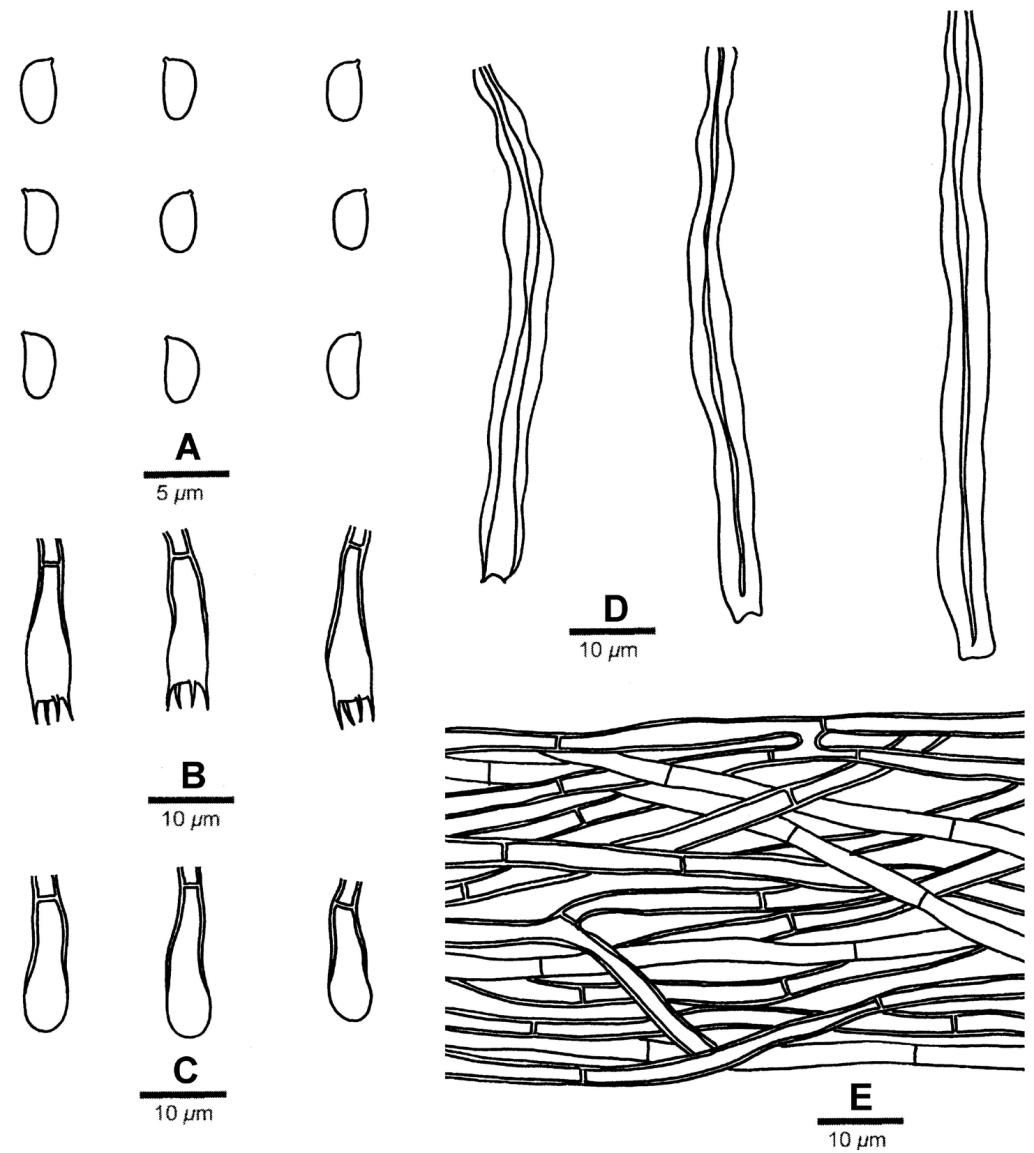
Microscopic structures of *Leifiabrevispora* (drawn from LWZ 20170820-46, holotype). A. basidiospores. B. basidia. C. basidioles. D. cystidia. E. subicular hyphae.

## Discussion

In this study, the newly generated ITS and 28S sequences were incorporated into the dataset of Larsson et al. (2006) and, in the resulting phylogeny (Figure 1), clades are labelled A-F as in Larsson et al. (2006). The differences of phylogeny observed between the current study and Larsson et al. (2006) might reflect that the ITS and 28S dataset itself is not enough to reliably resolve the relationships within Hymenochaetales. Similar to Larsson et al. (2006), *Leifia* formed a sister lineage to *Odonticium* with strong support in the current phylogeny (Figure 1). The five taxa of *Leifia* and the two of *Odonticium* were clearly separated and recovered as independent, fully supported clades. Morphologically, *Leifia* is well distinguished from *Odonticium* by its grandinioid hymenophore with hyphal strands, numerous thick-walled cystidia with an invaginated apical end and narrowly and thick-walled basidia (Eriksson et al. 1981). Therefore, we propose to resurrect *Leifia* as an independent genus in Hymenochaetales.

Amongst the four newly sequenced taxa in *Leifia* clade, *LWZ 20170820-46* and *LWZ 20170820-48* represent the new species *L.brevispora*, while *LWZ 20171015-36* and *LWZ 20171015-38*, both collected from Bidoup Nui Ba National Park, Da Lat, Viet Nam, seem to represent two undescribed taxa. *LWZ 20171015-36* differs from *L.brevispora* and *L.flabelliradiata* by fairly thick basidiocarps and *LWZ 20171015-38* differs by having basidia and basidioles that swell in KOH. Moreover, *LWZ 20171015-38* grows on fallen branches of *Pinus*, while the other three specimens were all collected from angiosperm substrates. Although the morphological characters of *LWZ 20171015-36* and *LWZ 20171015-38* are unique in *Leifia*, we feel more samples need to be examined before describing them as new species.

## Supplementary Material

XML Treatment for Leifia brevispora
